# Effects of a neo-functional psychotherapeutic approach on post-traumatic stress disorder following spontaneous coronary artery dissection: a case report

**DOI:** 10.3389/fcvm.2026.1821307

**Published:** 2026-09-03

**Authors:** Emilia Solinas, Filippo Luca Gurgoglione, Andrea Denegri, Giorgio Benatti, Rossella Giacalone, Manjola Noni, Giulia Magnani, Luigi Vignali, Giampaolo Niccoli, Caterina Viaro, Enrica Pedrelli, Rita Pinetti, Roberta Rosin, Luciano Sabella, Giuseppe Rizzi, Luciano Rispoli

**Affiliations:** 1 Department of Cardio-Thoraco-Vascular Medicine, Division of Cardiology Parma University Hospital, Parma, Italy; 2 Division of Cardiology, University of Parma, Parma University Hospital, Parma, Italy; 3 European School in Functional Psychotherapy (SEF), Padua, Italy; 4 Founder of European School in Functional Psychotherapy (SEF), Naples, Italy

**Keywords:** autonomic nervous system, case report, heart rate variability, neo-functional approach, post-traumatic stress disorder, psychocardiology, spontaneous coronary artery dissection

## Abstract

**Background:**

Post-traumatic stress disorder (PTSD) is increasingly recognized after acute coronary syndromes, including spontaneous coronary artery dissection (SCAD), a condition predominantly affecting women. PTSD symptoms may negatively influence autonomic regulation, adherence to therapy, and cardiovascular outcomes. Evidence regarding structured psychotherapeutic interventions targeting SCAD-related PTSD is limited.

**Case presentation:**

A 58-year-old woman was admitted with non–ST-elevation myocardial infarction due to SCAD involving the first obtuse marginal branch of the circumflex artery, managed conservatively. The event occurred one day after the sudden death of her son. Psychological evaluation revealed severe PTSD (Impact of Event Scale-Revised score: 57), characterized by hyperarousal, intrusive memories, avoidance, and sleep disturbance. The patient underwent a 12-month Neo-Functional Psychotherapeutic Approach integrated within cardiologic follow-up. The intervention included grounding techniques, diaphragmatic breathing, emotional processing, and cognitive restructuring.

**Results:**

At 6 months, psychometric reassessment showed a reduction in stress indices. Holter-based heart rate variability (HRV) demonstrated preserved vagal-related activity indices (SDNN 120–130 ms; RMSSD 38–40 ms). Clinically, intrusive symptoms and somatic hyperarousal progressively decreased, accompanied by improvement in social and daily functioning.

**Conclusion:**

This case suggests that integration of a structured body-oriented psychotherapeutic intervention within cardiologic care is feasible and may be associated with psychological improvement and favorable autonomic modulation in PTSD following SCAD. Larger prospective studies are warranted these preliminary observations.

## 1 Introduction

Spontaneous coronary artery dissection (SCAD) is a non-atherosclerotic uncommon cause of acute coronary syndrome (ACS), predominantly affecting women, particularly during the peri-menopausal period or in association with gender related non traditional cardiovascular risk factors (NTCVRF), including emotional or physical stressors. SCAD is increasingly recognized as an important underlying etiology of myocardial infarction with non-obstructive coronary arteries (MINOCA) when the dissection does not produce angiographically overt luminal stenosis; clinically, however, SCAD can present across the full spectrum of acute coronary syndromes, manifesting as ST-elevation myocardial infarction (STEMI) or non-ST-elevation myocardial infarction (NSTEMI) or cardiac arrest, depending on the degree and location of coronary flow compromise. The pathophysiology of SCAD identifies the formation of an intramural hematoma as its origin, or intimal disruption rather than atherosclerotic plaque rupture. Sex-specific factors due to fluctuations in estrogen levels, microvascular reactivity, and differences in autonomic regulation may amplify the impact of stress-induced sympathetic overactivation and inflammatory responses, thereby increasing vascular frailty. Reduced HRV in women following acute emotional trauma may further compromise the cholinergic anti-inflammatory pathway, reinforcing the neuro–immune–cardiovascular loop and contributing to heightened susceptibility to SCAD (1–4). Integrating sex-specific considerations into both mechanistic models and psychotherapeutic interventions may enhance risk stratification and recovery outcomes. Beyond its acute cardiovascular implications, SCAD also constitutes a psychologically traumatic event. PTSD symptoms—including intrusive recollections, hyperarousal, and avoidance—have been described following ACS and are associated with impaired quality of life, reduced adherence to secondary prevention, and increased risk of recurrent cardiovascular events (5). Of note high psychological distress following SCAD may exacerbate autonomic dysregulation, triggering a cascade of neuroendocrine and inflammatory responses that amplify cardiovascular risk (6). On the other hand the relationship between PTSD and the risk of adverse cardiac events has also been described (7–9). Reduced heart rate variability (HRV), a robust non-invasive marker of autonomic imbalance, has been consistently associated with heightened sympathetic activity, impaired parasympathetic modulation, systemic inflammation, and adverse cardiovascular outcomes in diverse coronary populations (10, 11). Observational studies further support that low HRV predicts major adverse cardiovascular events, emphasizing its role as a mechanistic link between stress, inflammation, and cardiovascular morbidity. Autonomic imbalance represents a plausible mechanistic pathway linking psychological distress and cardiovascular risk (12, 13). While mind–body interventions have demonstrated beneficial effects in coronary artery disease (14–18), evidence specifically addressing structured psychotherapy in SCAD-related PTSD is scarce. The Neo-Functional Psychotherapeutic Approach (NFPA) derives from Neo-Funzionalismo, a theoretical and clinical model developed in Italy from the 1970s onward by Luciano Rispoli within the tradition of body-oriented psychotherapy (Reich, Lowen, Boyesen). It conceptualizes the Self as an integrated organization of psycho-bodily functions present from birth, and draws on psychoneuroendocrinoimmunology (PNEI) to describe the organism as a self-regulated network spanning psychological, neurological, endocrine, and immune systems (Rispoli, 1993, 2008, 2016). Its central therapeutic construct, the Basic Experiences of the Self, addresses fundamental psycho-bodily needs through integrated body-based and verbal techniques, aiming to restore physiological, emotional, and cognitive regulation. This body–mind integrative orientation motivated its selection for a patient in whom a physical cardiac event (SCAD) and acute psychological trauma (sudden bereavement) were closely intertwined, and is consistent with our use of heart-rate variability as a psychophysiological outcome measure within an integrated cardiology–psychotherapy follow-up. Empirical outcome research on this approach remains limited (19, 20). Here, we report the case of a patient with PTSD who presented with Non-ST-elevation myocardial infarction due to SCAD, treated with a NFPA, focusing on psychological and autonomic outcomes.

## 2 Case presentation

A 58-year-old woman was admitted in August 2024 with non–ST-elevation myocardial infarction. Coronary angiography revealed SCAD of the first obtuse marginal branch of the circumflex artery without distal flow limitation; conservative management was adopted. Left ventricular ejection fraction was preserved, and no arrhythmic or hemodynamic complications occurred during hospitalization. Cardiovascular risk factors included hypertension, dyslipidemia, overweight status, and family history of cardiovascular disease. A major acute emotional stressor preceded the event, as the patient's 22-year-old son had died in a car accident the day before hospital admission.

For the psychological clinical assessment all the tools are listed in Table 1. Pre-Post treatment and 6 months results, including normative values are shown in Tables 2, 3 and 4.

**Table 1:** Psychological evaluation.

Assessment domain	Instrument	Timing
Psychometric tests	PSS-10, IES-R	PRE-POST TX
Autonomic/physiological stress index	MSP total stress index (24-h Holter HRV)	POST-TX
Sleep, social interaction, daily activities, social/occupational functioning, symptom trajectory between formal assessments)	Clinical interview	Each follow-up visit
Startle response/other psychophysiological markers	Not assessed	—

**Table 2:** Normative values of PSS-10 according to Cohen et al, 1983 and IES-R according to Creamer et al, 2003.

PSS-10		
Level of symptos	Pt score	Score
Normal		0–13
Moderate stress		14–26
Severe	34	27–40

IES-R		
Level of symptos	Pt score	Score
None		0–23
Mild symptoms concern for PTSD		24–32
Moderate symptoms Probable PTSD		33–37
Severe	57	38–88

**Table 3:** 6-month reassessment.

Parameter	Pre	Post
PSS-10	34	13
IES-R	57	36
MSP	NA	0.6

**Table 4:** Holter-based HRV analysis at 6 months.

Parameter	Value	Reference range
SDNN	120–130 ms	>100 ms
SDNN-I	70 ms	>40 ms
RMSSD	38–40 ms	>30 ms
Triangular index	30	>20

Validated Psychometric scales including 10-item Perceived Stress Scale (PSS-10) (21) and IES-R (21–24) together with the presence of clinical symptoms were assessed through semi-structured clinical interviews conducted by the treating clinician at each follow-up visit. Clinical features including intrusive recollections, avoidance behaviors, and negative alterations in mood, along with global distress were captured by the IES-R and PSS at pre and post-treatment time points;

The MSP total stress ind. ex was captured only post-treatment. The MSP (Mesure du Stress Psychologique) is a multidimensional instrument assessing self-reported and behaviorally observed indicators of psychological stress, originally developed by Lemyre, Tessier, & Fillion (1990) and adapted for Italian use by Di Nuovo, Rispoli, & Genta (2000) (25–27).

Signs of marked somatic sympathetic hypertonus were identified through combined clinical assessment:

1. direct clinical/bodily observation of muscular tone, posture, breathing pattern, and movement by the treating clinician, consistent with the body-oriented assessment framework of the NFPA (Di Nuovo, Rispoli, & Genta, 2000) (27), noting increased resting muscular tension in several body districts like neck, shoulders, back, legs and arms,
2. clinical signs of autonomic sympathetic activation/reduced vagal tone, including resting tachycardia, arterial hypertension, diaphoresis, tremor, assessed by the treating clinicians at baseline during the acute phase

All other clinical features (e.g., sleep quality, social/occupational functioning, symptom trajectory between formal assessments) were assessed through semi-structured clinical interview by the treating clinician at each follow-up visit. No psychophysiological testing (e.g., startle response) was performed.

Acute Post-event psychological assessment revealed:

- Perceived Stress Scale (PSS-10): 34
- Impact of Event Scale-Revised (IES-R): 57

Acute Post-event semi-structured clinical interviews evidenced: hypervigilance, intrusive memories, exaggerated startle response, avoidance behaviors, emotional detachment, sleep disturbance, and persistent somatic tension

The patient's baseline IES-R score of 57 therefore corresponds to severe symptoms of PTSD. The patient's baseline PSS-10 score of 34 corresponds to severe stress symptoms.

The findings were consistent with PTSD according to DSM-5 criteria and to the Neo-Functional diagnostic chart.

Screening for extracoronary vascular abnormalities identified a focal pseudoaneurysm of the left internal carotid artery. Post-event coronary CT analysis showed increased pericoronary fat attenuation index (pFAI) values suggestive of enhanced coronary inflammation, exceeding the established normal threshold of -70 Hounsfield units.

The patient entered a structured cardiologic follow-up and began a weekly Neo-Functional Psychotherapeutic program lasting 12 months (September 2024–December 2025).

## 3 Intervention

The Neo-Functional Psychotherapeutic Approach is an integrative model addressing bodily, emotional, and cognitive processes, with particular emphasis on autonomic regulation (28, 29).

The intervention followed a structured protocol adapted for ongoing trauma and included:

- Grounding and proprioceptive exercises
- Diaphragmatic breathing aimed at vagal modulation
- Guided emotional processing
- Progressive cognitive reframing of trauma-related meaning
- Body scan techniques to identify and reduce residual somatic activation

Initial sessions prioritized stabilization and restoration of perceived safety before gradual trauma-related processing. Therapy was delivered weekly for 12 months in conjunction with standard cardiologic management.

## 4 Outcomes

### 4.1 Psychometric outcomes

At 6 months follow up psychological assessment by means of PSS-10, IES-R and MSP (19, 20, 25) revealed:

- SP Total Stress Index: 0.6 (moderate range) (25–27).
- Reduction in clusters related to irritability, psycho-physiological activation, and depressive anxiety

At serial follow-up clinical interviews, the patient reported—and the treating clinician observed—a progressive decrease in intrusive recollections and avoidance behaviors. The patient also reported having resumed quite regular social interaction and daily activities.

The patient resumed regular social interaction and daily activities.

### 4.2 Autonomic assessment

These findings indicate preserved overall HRV and relative predominance of vagal-related indices, consistent with autonomic flexibility and according to values derived from twenty-four hour time domain heart rate variability and heart rate adjusted to age and gender (30). We noted that these values are also above the heart-rate-corrected median reported by van den Berg et al. (2018) for women aged 50–59; however, those normative values were derived from 10-s ECGs, corrected for heart rate, in a cardiologically healthy cohort excluding beta-blocker users, and are therefore not directly comparable in either magnitude or correction status to our uncorrected 24-hour Holter indices.

No recurrent ischemic events or arrhythmic complications occurred during follow-up.

## 5 Discussion

This case highlights a clinically relevant intersection between SCAD, psychological trauma, and autonomic regulation.

### 5.1 SCAD as a stress-related vascular event

SCAD predominantly affects women and is frequently preceded by intense emotional or physical stressors. Acute stress activates the sympathetic nervous system and the hypothalamic–pituitary–adrenal (HPA) axis, leading to catecholamine surge, blood pressure variability, endothelial dysfunction, and inflammatory activation. These mechanisms may increase shear stress and impair vascular integrity, potentially contributing to arterial wall vulnerability in predisposed individuals (1–4).

PTSD and trauma exposure have been independently associated with increased cardiovascular risk, particularly among women (7–9). In the present case, the temporal proximity between sudden bereavement and SCAD onset suggests a potential stress-triggered mechanism. Although causality cannot be established, this observation is consistent with current evidence linking emotional triggers to SCAD occurrence and cardiovascular events.

### 5.2 Autonomic imbalance, HRV, and inflammation

HRV is a validated non-invasive marker of autonomic nervous system balance (30, 31). Reduced HRV has been associated with increased risk of cardiac mortality and adverse cardiovascular outcomes (6). Lower vagally mediated indices, such as RMSSD, reflect diminished parasympathetic modulation and have been linked to cardiovascular risk factors (10, 11).

The biological plausibility of this association is supported by the “inflammatory reflex” described by Tracey, in which vagal efferent activity modulates cytokine production through the cholinergic anti-inflammatory pathway. Subsequent work has demonstrated that HRV may serve as a surrogate marker of this neuroimmune interaction. Reduced vagal tone has been associated with increased inflammatory burden, endothelial dysfunction, and progression of cardiovascular disease (14–17).

SCAD has been associated with inflammatory activation and vascular vulnerability (1–4). In our patient, coronary CT analysis suggested increased pericoronary inflammatory activity. At 6-month follow-up, HRV indices (SDNN and RMSSD) were within normal-to-high ranges, indicating preserved autonomic flexibility. Although baseline HRV data were unavailable, the observed profile is consistent with relative vagal predominance and reduced sympathetic overactivation.

### 5.3 PTSD, autonomic dysregulation and cardiovascular risk

PTSD is characterized by chronic hyperarousal and sustained sympathetic activation. Meta-analytic evidence demonstrates that PTSD is associated with reduced HRV, particularly in vagally mediated components (32). Twin studies have further confirmed impaired autonomic modulation in individuals with PTSD, independent of shared genetic and environmental factors (33).

These autonomic alterations may partly explain the increased incidence of cardiovascular events observed in patients with PTSD (7–9). Persistent sympathetic predominance, impaired baroreflex sensitivity, and systemic inflammation represent plausible mediators linking psychological trauma to cardiovascular pathology (10, 11).

In the present case, severe PTSD symptoms were initially accompanied by marked somatic hypertonus and hypervigilance, consistent with sympathetic hyperactivation. The Neo Functional psychotherapeutic intervention emphasized diaphragmatic breathing and interoceptive awareness—techniques known to influence vagal modulation and HRV (14–18). At follow-up, improvement in stress indices and preservation of vagally mediated HRV parameters were observed.

While spontaneous recovery and natural autonomic adaptation cannot be excluded, the physiological coherence between symptom improvement and autonomic markers supports the hypothesis of a potential psychocardiologic interaction.

### 5.4 Toward an integrated psychocardiologic model

Interventions targeting autonomic regulation, including structured stress-management programs, HRV biofeedback, and mind–body approaches, have demonstrated improvements in HRV, baroreflex sensitivity, and myocardial stress responses, indicating that enhancing autonomic flexibility may mitigate the physiological consequences of psychological distress (16–18). These interventions also reduce anxiety and depressive symptoms and may attenuate systemic inflammation, suggesting that integrated psychophysiologic strategies can modulate the neuro–immune–cardio axis in coronary disease. While such evidence is robust in general coronary populations, data specific to SCAD-associated PTSD remain scarce (6), highlighting the need for tailored psychotherapeutic approaches that address both psychological and autonomic dysregulation.

In the present case, we applied a 12-month NFPTA (28, 29), focusing on restoration of bodily, emotional, and cognitive integration. Clinical outcomes demonstrated improvements in both psychometric measures of PTSD and autonomic function, evidenced by enhanced HRV indices, including SDNN and RMSSD, consistent with increased parasympathetic activation. These findings support a plausible mechanistic pathway in which NFPTA facilitates rebalancing of the autonomic nervous system, reduces inflammatory stress responses, and promotes cardiovascular recovery in SCAD patients with PTSD. The case also underscores the potential importance of sex-specific vulnerability, as SCAD predominantly affects women, often in perimenopausal or stress-sensitive contexts, suggesting that targeted interventions addressing autonomic and psychophysiologic mechanisms may be particularly beneficial in this population.

This case supports a conceptual framework in which:

1. Acute emotional stress contributes to autonomic imbalance and inflammatory activation
2. PTSD-related autonomic dysregulation may increase cardiovascular vulnerability
3. NFPTA, targeting vagal modulation, may influence stress-related autonomic pathways and potentially inflammatory tone.

This model remains hypothetical and requires validation in prospective controlled studies including longitudinal HRV monitoring, inflammatory biomarkers, and clinical endpoints in SCAD populations.

Taken together, this evidence positions NFPTA as a promising integrative strategy for PTSD management post-SCAD, bridging psychological recovery with neuro-autonomic modulation and reinforcing the relevance of addressing mind–body coherence to optimize cardiovascular outcomes.

## 6 Limitations

- Single-case design
- Absence of baseline HRV and MSP test prior to psychotherapy
- Lack of control condition
- Limited long-term follow-up
- Use of instruments not widely adopted in cardiovascular research
- Normative comparisons for HRV indices are complicated by heterogeneity across studies in recording duration, heart-rate correction method, and reference population; because our values are uncorrected 24-hour indices, comparisons to heart-rate-corrected, short-duration normative data should be interpreted with caution.
- Furthermore the reduction in intrusive recollections and avoidance behaviors, as well as the resumption of social interaction and daily activities, were assessed through semi-structured clinical interviews conducted by the treating clinician at each follow-up visit, rather than through administration of standardized psychometric instruments. This is consistent with routine clinical practice within the integrated cardiology–psychotherapy follow-up described in this case report; however we agree it does not provide a quantifiable change score comparable to the PSS-10, IES-R, or MSP indices. Future case reports ore case series would benefit from validated psychosocial/occupational functioning scales administered at every follow-up time point.

Prospective controlled studies are needed to evaluate reproducibility and clarify mechanistic pathways.

## 7 Conclusion

In this patient with PTSD following SCAD, integration of a structured Neo-Functional Psychotherapeutic Approach within cardiologic follow-up was feasible and associated with psychological improvement and preserved autonomic modulation.

These findings support further investigation of integrated psychocardiology models in women with SCAD and trauma-related symptoms.

## Data Availability

The raw data supporting the conclusions of this article will be made available by the authors, without undue reservation.
